# Regulation of epileptiform activity by two distinct subtypes of extrasynaptic GABA_A_ receptors

**DOI:** 10.1186/1756-6606-6-21

**Published:** 2013-05-01

**Authors:** Yajie Sun, Zheng Wu, Shuzhen Kong, Dongyun Jiang, Anar Pitre, Yun Wang, Gong Chen

**Affiliations:** 1 Institutes of Brain Science and State Key Laboratory for Medical Neurobiology, Fudan University, Shanghai, 200032, China; 2 Department of Biology, Huck Institutes of Life Sciences, The Pennsylvania State University, University Park, PA, 16802, USA

**Keywords:** Extrasynaptic GABA_A_ receptor, α5 subunit, δ subunit, Tonic inhibition, Epileptogenesis, Epileptiform activity, THIP, Seizure behavior

## Abstract

**Background:**

GABAergic deficit is one of the major mechanisms underlying epileptic seizures. Previous studies have mainly focused on alterations of synaptic GABAergic inhibition during epileptogenesis. Recent work suggested that tonic inhibition may also play a role in regulating epileptogenesis, but the underlying mechanism is not well understood.

**Results:**

We employed molecular and pharmacological tools to investigate the role of tonic inhibition during epileptogenesis both *in vitro* and *in vivo*. We overexpressed two distinct subtypes of extrasynaptic GABA_A_ receptors, α5β3γ2 and α6β3δ receptors, in cultured hippocampal neurons. We demonstrated that overexpression of both α5β3γ2 and α6β3δ receptors enhanced tonic inhibition and reduced epileptiform activity *in vitro*. We then showed that injection of THIP (5 μM), a selective agonist for extrasynaptic GABA_A_ receptors at low concentration, into rat brain also suppressed epileptiform burst activity and behavioral seizures *in vivo*. Mechanistically, we discovered that low concentration of THIP had no effect on GABAergic synaptic transmission and did not affect the basal level of action potentials, but significantly inhibited high frequency neuronal activity induced by epileptogenic agents.

**Conclusions:**

Our studies suggest that extrasynaptic GABA_A_ receptors play an important role in controlling hyperexcitatory activity, such as that during epileptogenesis, but a less prominent role in modulating a low level of basal activity. We propose that tonic inhibition may play a greater role under pathological conditions than in physiological conditions in terms of modulating neural network activity.

## Background

Many antiepileptic drugs are targeting GABAergic synaptic transmission, but may cause certain side effects [1,2]. GABA_A_ receptors (GABA_A_-Rs) are abundant not only at synaptic sites, but also at extrasynaptic sites. Synaptic GABA_A_-Rs have low affinity for GABA, are activated in a transient manner by GABA released form presynaptic vesicles, and primarily mediate phasic inhibitory transmission. In contrast, extrasynaptic GABA_A_-Rs exhibit high affinity for GABA, are persistently activated by low concentration of ambient GABA, and mediate tonic inhibition [3-5]. There are two distinct subtypes of extrasynaptic GABA_A_-Rs in the brain, one contains the α5 subunit [6-10] and the other contains the δ subunit [11-13]. The α5-GABA_A_-Rs are sensitive to a specific inverse agonist L-655,708 [14,15], while the δ-GABA_A_-Rs are typically insensitive to benzodiazepine [16,17] but highly sensitive to THIP (gaboxadol) [18,19].

Functional deficit of synaptic GABAergic inhibition plays an important role in the etiology of epilepsy [2,20-22]. Recent studies revealed a possible role of tonic inhibition in modulating epileptic seizures [23,24]. A significant reduction of GABA_A_-R α5 and δ subunit level has been reported in the hippocampus of animals with temporal lobe epilepsy (TLE) [8,25,26]. Interestingly, the decrease of δ subunit may be compensated by an increase of α4 and γ2 subunits [26-28]. Mutations in the δ subunit of GABA_A_-Rs have been mapped in human epilepsy patients [29,30]. Increased δ subunit level during diestrus stage of ovarian cycle has been associated with less seizure activities in kainic acid-induced epilepsy models [31]. However, in the pyramidal neurons of hippocampal CA1 region, the α5 GABA_A_-R mediated tonic current was reduced but overall tonic inhibition was not changed or even increased in pilocarpine epilepsy model [32]. Furthermore, enhanced tonic inhibition in thalamocortical neurons was reported to induce absence seizure [33], suggesting that different tonic inhibition may play different roles in different brain regions.

Here we investigated the functional role of two distinctly different subtypes of extrasynaptic GABA_A_-Rs in hippocampal epileptogenesis. We demonstrated that enhancing tonic inhibition by overexpressing either the α5β3γ2 or α6β3δ extrasynaptic GABA_A_ receptors significantly inhibited the formation of epileptiform activity in hippocampal cultures. Furthermore, *in vivo* injection of selective extrasynaptic GABA_A_-R agonist THIP also inhibited epileptiform bursting activity in anesthetized rats and seizure behaviors in freely moving rats. Interestingly, low concentration of THIP did not affect basal level of neuronal activity, but significantly suppressed higher frequency neuronal firing. Therefore, our data suggest that tonic inhibition mediated by extrasynaptic GABA_A_-Rs may play a more prominent function in pathological conditions such as during epileptogenesis.

## Results

### Molecular enhancement of tonic GABA currents after overexpressing α5β3γ2 GABA_A_ receptors

**Figure 1:**
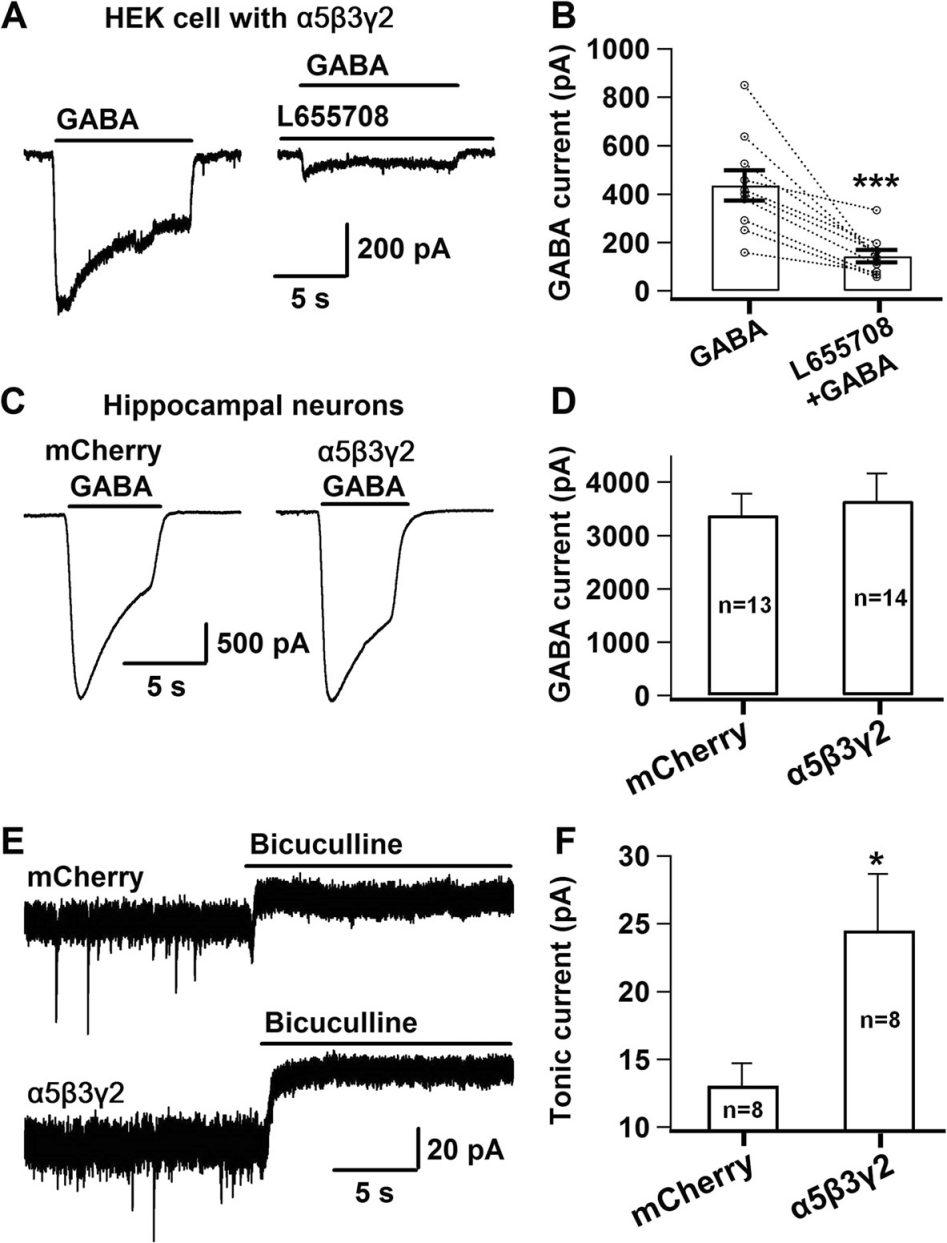
**Tonic GABA current increased after the overexpression of α5β3γ2 GABA**_**A **_**receptors. A**, Typical GABA (100 μM) induced currents in HEK293T cells transfected with α5β3γ2 subunits (left panel), which could be largely blocked by α5 subunit-specific inverse agonist L655,708 (100 nM, right panel). **B**, Summarized data showing GABA-induced α5β3γ2 receptor currents in HEK293T cells significantly inhibited by L655,708 (Control, 437.1 ± 63.1 pA, n = 10; L655,708, 142.8 ± 25.7 pA, n = 10; ***, p < 0.001). **C**, Typical GABA current traces in cultured hippocampal neurons transfected with mCherry or plus the α5β3γ2 subunits. **D**, Bar graphs showing no significant difference between the total whole-cell GABA currents in neurons transfected with mCherry or plus the α5β3γ2 subunits. **E**, Representative tonic GABA currents revealed by rapid application of GABA_A_-R blocker bicuculline (100 μM) in hippocampal neurons transfected with mCherry or plus the α5β3γ2 subunits. **F**, Summarized data showing that tonic GABA current in α5β3γ2-transfected neurons (24.5 ± 4.2 pA, n = 8) was significantly increased in comparison with the control neurons (13.1 ± 1.7 pA, n = 8; *, p < 0.05).

We have previously demonstrated that epileptiform activity downregulates tonic inhibition mediated by extrasynaptic GABA_A_ receptors [34]. In this study, we investigated the effect of enhanced tonic inhibition on epileptiform activity. The majority of extrasynaptic GABA_A_ receptors (GABA_A_-Rs) can be categorized into two distinct subtypes, containing either the α5 or the δ subunit. The α5-GABA_A_-Rs are mainly expressed in the hippocampus of adult brain, while δ-GABA_A_-Rs are mostly expressed in dentate and cerebellar granule cells as well as in the thalamus. To understand which subtype of extrasynaptic GABA_A_-Rs may affect epileptiform activity, we overexpressed both the α5β3γ2 and α6β3δ GABA_A_-Rs in hippocampal neurons to enhance tonic inhibition and tested their effects on epileptiform activity. Since α5 subunit is normally expressed in hippocampal pyramidal neurons [6], we first tested the effect of α5β3γ2 receptors on epileptiform activity. We have tested several α5 subunits and found a right one that can give large GABA-evoked current when coexpressed with the β3 and γ2 subunits in HEK 293T cells (Figure 1A). The inverse agonist L-655,708 (100 nM) specific for the α5 subunit significantly inhibited the GABA-evoked currents (Figure 1A-B; Control, 437 ± 63 pA, n = 10; L-655,708, 143 ± 26 pA, n = 10; ***, p < 0.001; Student’s *t* test), confirming that the GABA current was mediated by α5 subunit-containing GABA_A_-Rs. We next overexpressed the α5β3γ2 receptors in cultured hippocampal pyramidal neurons. The GABA-evoked whole-cell currents appeared to be similar between neurons transfected with the α5β3γ2 receptors and the mCherry for control (Figure 1C-D; mCherry, 3380 ± 408 pA, n =13; α5β3γ2, 3646 ± 518 pA, n = 14; p > 0.5). However, tonic GABA currents revealed by acute application of GABA_A_-R blocker bicuculline (Bic, 40 μM; together with 1 μM TTX and 10 μM DNQX) [9,34,35], were significantly increased in α5β3γ2-transfected neurons compared to mCherry-transfected control neurons (Figure 1E-F; mCherry, 13.1 ± 1.7 pA, n = 8; α5β3γ2, 24.5 ± 4.2 pA, n = 8; p < 0.05). These data demonstrated that overexpression of the α5β3γ2 receptors enhanced tonic GABA currents in hippocampal neurons.

### Tonic inhibition mediated by the α5β3γ2 receptors suppresses epileptiform activity

**Figure 2:**
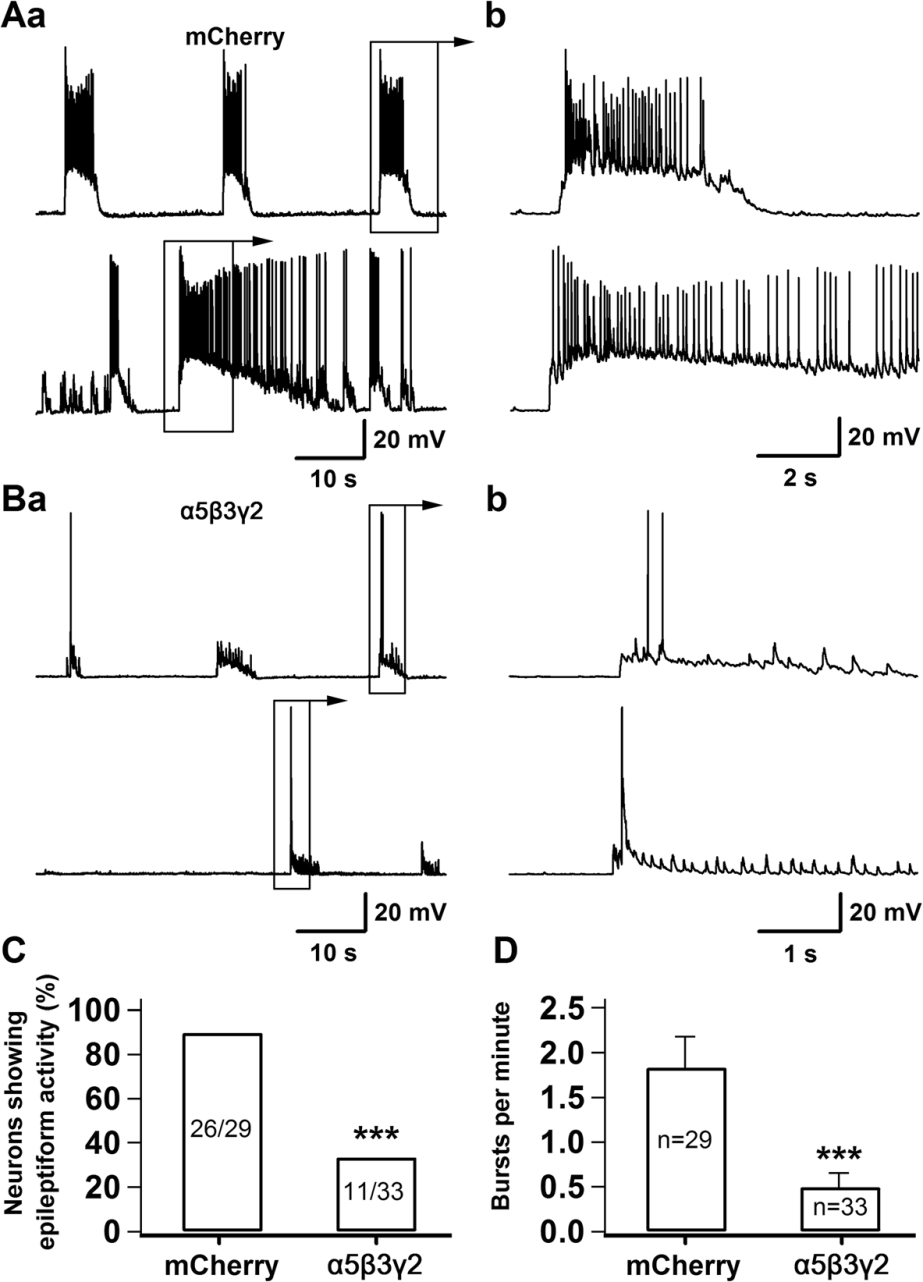
**Inhibition of epileptiform activity in cultured hippocampal neurons overexpressing α5β3γ2 receptors. A**, Typical traces from two hippocampal neurons showing the epileptiform burst activity after chronic CTZ treatment (5 μΜ, 24 h). Panel b shows the expanded view of a single epileptiform burst from panel a. Epileptiform burst is characterized by a train of action potentials on a large depolarization shift. **B**, Representative traces showing the lack of epileptiform bursts in two hippocampal neurons transfected with the α5β3γ2 receptors. **C**, Bar graphs illustrating that overexpression of α5β3γ2 receptors significantly reduced the percentage of neurons showing epileptiform activity after chronic CTZ treatment (mCherry, ~90%, n = 29; α5β3γ2, ~33%, n = 33; ***, p < 0.001). **D**, Neurons transfected with α5β3γ2 receptors showing lower burst frequency (0.49 ± 0.16 per min, n = 33), compared to mCherry controls after CTZ treatment (1.83 ± 0.35 per min, n = 29; ***, p < 0.001).

We further tested whether epileptiform activity was affected by the enhanced tonic GABA current in neurons overexpressing the α5β3γ2 receptors. We previously established a unique cyclothiazide (CTZ) induced epilepsy model both *in vitro* and *in vivo*[36-39]. The advantage of CTZ model is its reliability in inducing epileptiform activity without significant cell death [36]. We treated hippocampal culture with CTZ (5 μΜ, 24 hr) to induce epileptiform activity, similar to reported before [36]. Control neurons transfected with mCherry showed robust epileptiform activity after CTZ-treatment, with a train of high-frequency action potentials overlaying on the plateau of large depolarization shifts (Figure 2A). In contrast, the majority of neurons transfected with the α5β3γ2 receptors did not show clear epileptiform activity, except some sparse action potentials on top of small depolarization bursts (Figure 2B). Quantitatively, about 90% of control neurons showed epileptiform activity after CTZ-treatment (26 out of 29 neurons), whereas only about 33% of neurons transfected with the α5β3γ2 receptors showed epileptiform activity (11 out of 33 neurons) (Figure 2C). The average frequency of epileptiform bursts was also significantly reduced in α5β3γ2-transfected neurons (Figure 2D; mCherry, 1.83 ± 0.35 per min, n = 29; α5β3γ2, 0.49 ± 0.16 per min, n = 33; p < 0.001). Together, our experiments demonstrated that enhanced tonic inhibition mediated by the α5β3γ2 receptors significantly suppressed the formation of epileptiform activity in hippocampal neurons.

### Inhibition of epileptiform activity by the α6β3δ receptors

**Figure 3:**
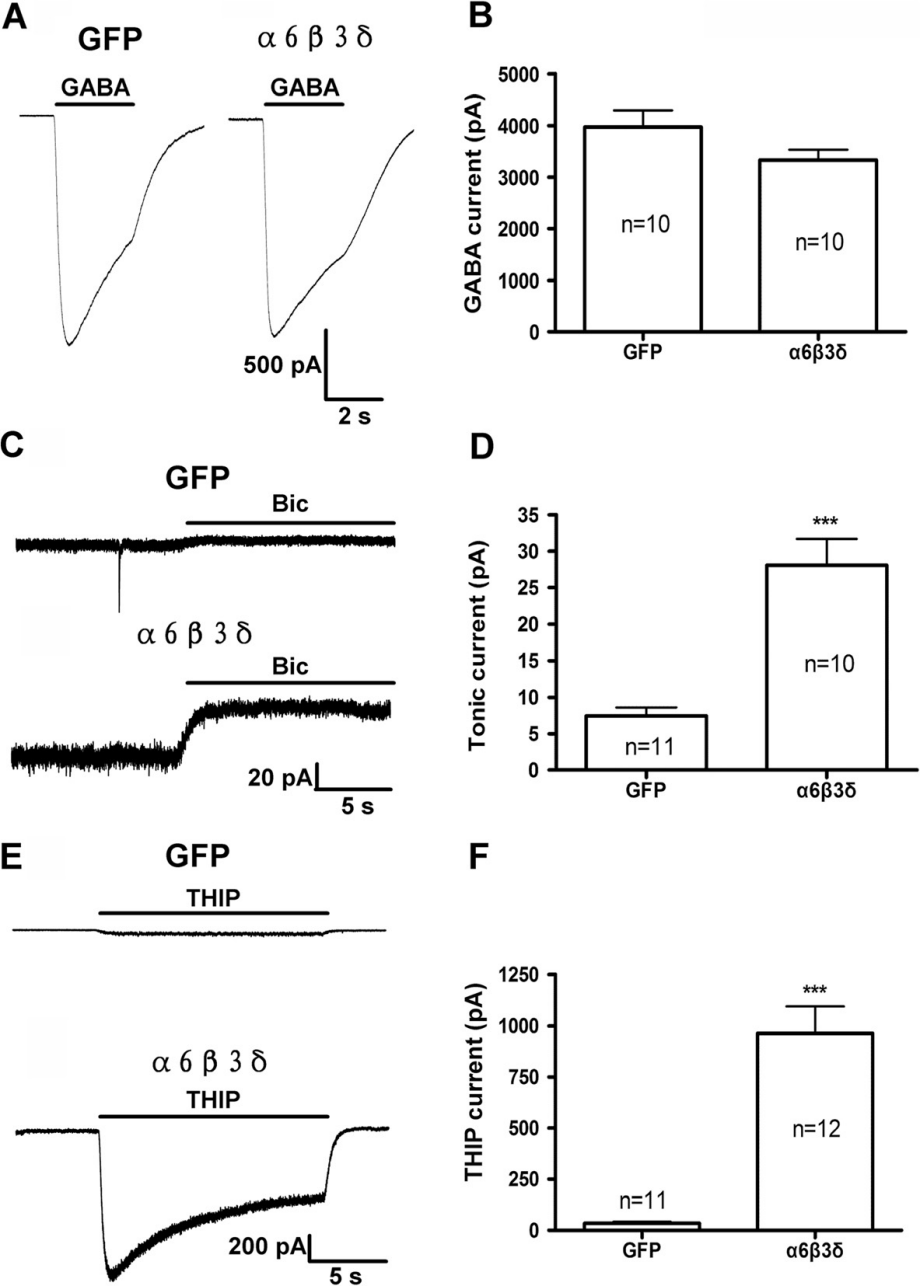
**Overexpression of α6β3δ subunits results in large tonic GABA current in cultured hippocampal neurons. A**, Typical recordings showing whole-cell currents induced by rapid application of GABA (20 μM) in GFP control and α6β3δ-transfected neurons in the presence of TTX (1 μM) and DNQX (10 μM). **B**, Summarized data showing no significant difference in whole-cell GABA currents between the two groups. **C**, Typical traces of tonic GABA currents, revealed by application of Bic (40 μM) in the presence of TTX (1 μM) and DNQX (10 μM), recorded from a GFP control neuron and a α6β3δ-transfected neuron. Holding potential = −70 mV. **D**, Summarized data showing that the average amplitude of tonic GABA currents was significantly increased in α6β3δ-transfected neurons (28.1 ± 3.6 pA, n = 10), compared to the GFP controls (7.4 ± 1.1 pA, n = 11; ***, *p* < 0.0001). **E**, Tonic currents activated by THIP (5 μM) in GFP control and α6β3δ-transfected neurons. **F**, Summarized data showing a significant increase of THIP-induced tonic currents after transfection of α6β3δ subunits (962 ± 130 pA, n = 12), compared to the GFP controls (35.3 ± 7.4 pA, n = 11; ***, *p* < 0.0001).

We next examined a distinctly different subtype of extrasynaptic GABA_A_-Rs, the α6β3δ receptors [11,12], in the regulation of epileptiform activity. We first examined whole-cell GABA currents after overexpressing α6β3δ receptors in hippocampal cultures. GABA-evoked whole-cell currents showed no difference between GFP- and α6β3δ-transfected neurons (Figure 3A). Quantitatively, the average GABA-evoked current in α6β3δ-transfected neurons was 3327 ± 206 pA (n = 10), which was not significantly different from the GFP control (3967 ± 328 pA, n = 10, *p* > 0.1; Figure 3B). To confirm functional expression of the α6β3δ receptors in hippocampal neurons, we examined tonic GABA current after Bic treatment and found that the tonic GABA current was greatly increased in α6β3δ-transfected neurons (Figure 3C). Quantitatively, the average amplitude of tonic GABA currents in α6β3δ-transfected neurons was 28.1 ± 3.6 pA (n = 10), significantly larger than that of control neurons (7.4 ± 1.1 pA, n = 11, *p* < 0.0001) (Figure 3D). Furthermore, we employed THIP (5 μM), a relatively specific agonist for δ subunit containing GABA_A_-Rs at low concentration, to investigate tonic currents in control and α6β3δ-transfected neurons. THIP activated a small non-desensitizing tonic current in GFP-transfected neurons, accompanied with an increase of the baseline noise (Figure 3E, top trace). In α6β3δ-transfected neurons, however, the THIP-induced tonic current was markedly increased (Figure 3E, bottom trace). The average amplitude of THIP current in control neurons was 35.3 ± 7.4 pA (n = 11), and greatly increased to 962 ± 130 pA in α6β3δ-transfected neurons (n = 12, *p* < 0.0001; Figure 3F). Thus, overexpression of α6β3δ receptors in hippocampal neurons significantly increased tonic GABA currents, similar to the overexpression of α5β3γ2 receptors.

**Figure 4:**
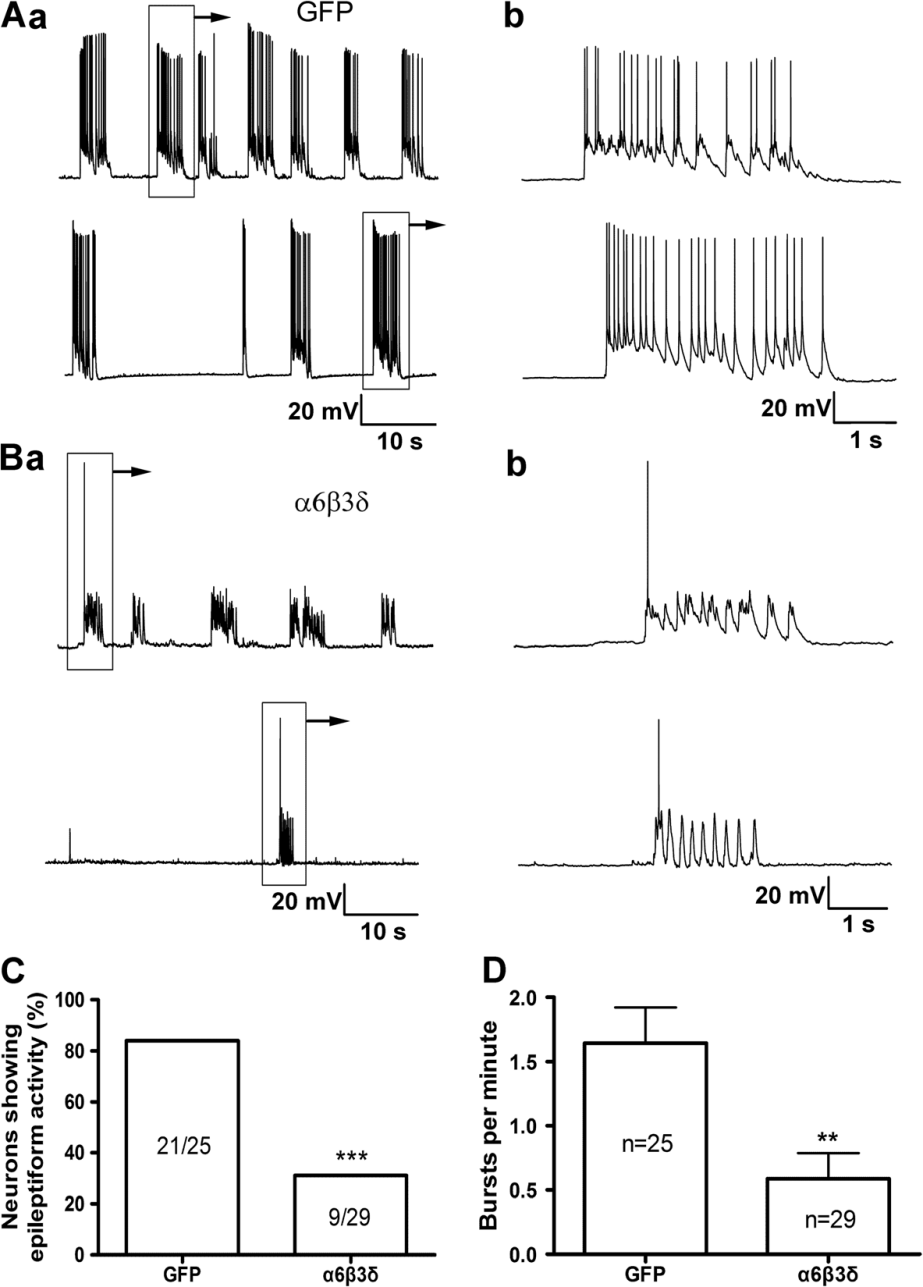
**Overexpression of α6β3δ receptors inhibits epileptiform bursting activity in cultured hippocampal neurons. Aa**, Representative traces showing the typical recurrent epileptiform bursts after chronic pretreatment with CTZ (5 μM, 24 h) in two different GFP-transfected pyramidal neurons. **Ab**, A single epileptiform burst in (**a**) was expanded to show a train of action potentials overlaying on a large depolarization shift. **Ba**, Representative traces showing the lack of typical epileptiform bursts in two α6β3δ-transfected hippocampal neurons. **Bb**, Expanded view of the boxed activity in (**a**). **C**, Bar graph showing the percentage of neurons with epileptiform bursting activity after chronic treatment with CTZ (5 μM for 24 h). *** *p* < 0.001, Pearson Chi-Square test. **D**, Bar graph showing a significant reduction of the average epileptiform burst frequency in neurons transfected with α6β3δ receptors, comparing to GFP controls after CTZ treatment. ** *p* < 0.01.

We then examined whether overexpression of the α6β3δ receptors has any effect on epileptiform activity. In GFP-transfected control neurons, CTZ-treatment (5 μΜ, 24 hr) induced robust epileptiform activity as expected (Figure 4A). In contrast, hippocampal neurons transfected with α6β3δ receptors showed a significant attenuation of epileptiform activity (Figure 4B). Quantitatively, the percentage of neurons displaying epileptiform activity was 84% (21 out of 25) in GFP-transfected controls, but reduced to 31% (9 out of 29) in α6β3δ-transfected neurons (Figure 4C; *p* < 0.001). Furthermore, the frequency of epileptiform bursts in α6β3δ-transfected neurons also reduced significantly (0.56 ± 0.2 bursts per min, n = 29, *p* < 0.003), compared to that in GFP control neurons (1.64 ± 0.28 bursts per min, n = 25) (Figure 4D). We investigated whether the overexpression of extrasynaptic GABA_A_-Rs would alter neuronal intrinsic properties, but found no changes in resting membrane potential (mCherry, -52.3 ± 1.3 mV, n = 15; α5β3γ2, -50.1 ± 1.6 mV, n = 12; α6β3δ, -51.5 ± 1.4 mV, n = 10; p > 0.5, one way ANOVA), membrane resistance (mCherry, 204 ± 22 MΩ, n = 15; α5β3γ2, 177 ± 17 MΩ, n = 12; α6β3δ, 237 ± 24 MΩ, n = 10; p > 0.4), or membrane capacitance (mCherry, 96.3 ± 7.8 pF, n = 15; α5β3γ2, 114.6 ± 10.8 pF, n = 12; α6β3δ, 95.7 ± 16.1 pF, n = 10; p > 0.19) in different transfected groups. Together, our data demonstrated that both α6β3δ and α5β3γ2 extrasynaptic GABA_A_-Rs have an important role in regulating hippocampal epileptogenesis *in vitro*.

### Tonic inhibition on *in vivo* epileptic seizures

**Figure 5:**
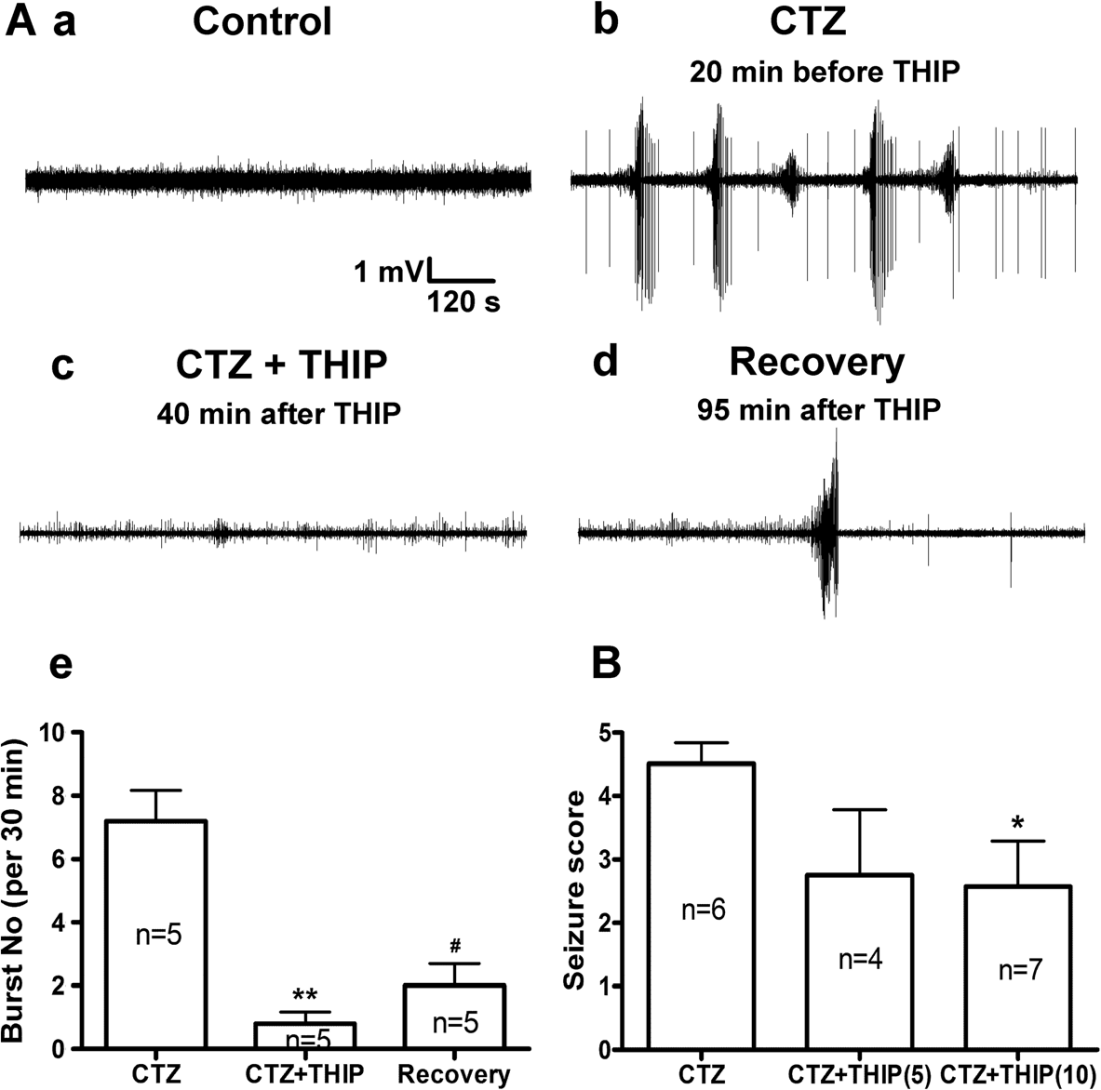
**THIP inhibits CTZ-induced epileptiform activity and seizure behavior. Aa-d**, Typical traces showing ‘silent’ baseline activity of hippocampal CA1 neurons in control condition (**a**), synchronized epileptiform bursting activities induced by CTZ (5 μmol) (**b**), inhibition of THIP (4 mg /kg) on burst activity (**c**), and the recovery after the THIP injection (**d**). A**e**, Bar histogram showing group data of THIP inhibition on CTZ-induced epileptiform burst activities. **B**, Bar histogram showing group data of CTZ-induced (5 μmol) seizure behavioral score and its significant attenuation by pre-treatment with THIP (10 mg/kg). THIP(5): 5 mg/kg THIP; THIP(10): 10 mg/kg THIP. * *p* < 0.05 and ** *p* < 0.01 in comparison with CTZ injection alone; # *p* < 0.05 for recovery, in comparison with CTZ + THIP.

After performing *in vitro* studies, we decided to further investigate the effect of tonic inhibition on epileptiform activity in *in vivo* condition. We recorded field potentials in the hippocampal CA1 pyramidal layer from 5 urethane-anaesthetized rats. In all 5 rats studied, the evoked responses following low frequency stimulation of CA3 region consisted of a large EPSP and a single population spike (PS) during control recordings (data not shown, see [36]), and the baseline activity was virtually ‘silent’ (Figure 5Aa). Following intra-cerebroventricular (i.c.v.) injection of CTZ (5 μmol in 5 μl), the single-peaked PS gradually transformed into a multiple-peaked event and the spontaneous recurrent epileptiform bursts were stable for at least 30 min in all 5 rats tested (Figure 5Ab). To enhance tonic inhibition *in vivo*, we chose to use agonist specific for extrasynaptic GABA_A_-Rs. For the α5 subunit-containing GABA_A_-Rs, there is no specific agonist available except inverse agonists that reduce receptor responses. However, THIP at low concentration is a selective agonist for the δ subunit-containing GABA_A_-Rs and widely used to elicit tonic inhibition both *in vitro* and *in vivo*[40-43]. Therefore, we delivered THIP (4 mg/kg) through the cannula pre-implanted in the lateral tail vein after the induction of epileptiform activity. Compared with the epileptiform burst number (7.2 ± 1.0 per 30 min) before THIP injection, the mean burst number after THIP administration was significantly reduced to 0.8 ± 0.4 (*p* < 0.003) over a 30 min analysis period (Figure 5Ac). Interestingly, the inhibitory effect of THIP on epileptiform activity was reversible, as indicated by a gradual increase of the mean burst number (2.0 ± 0.7 per 30 min) during a prolonged recovery period after the THIP injection (Figure 5Ad). The quantification of the burst frequency of CTZ, CTZ + THIP, and the recovery group was summarized in Figure 5Ae. These results indicate that acute application of THIP in *in vivo* condition can effectively suppress epileptiform activity.

Besides epileptiform activity, we further studied whether THIP can directly modulate CTZ-induced seizure behavior in freely moving rats [38]. CTZ was injected repeatedly each day (0.25 μmol i.c.v. for 3 consecutive days, total dose of 0.75 μmol) to induce seizure behavior without or with a pre-injection of THIP. The administration of THIP (5 or 10 mg/kg, i.p.) at 10 min before CTZ injection dose-dependently attenuated the convulsant seizures induced by CTZ. The seizure score was 4.5 ± 0.3 (n = 6) after CTZ injection alone, and significantly reduced to 2.6 ± 0.7 (n = 7) in THIP-preinjected (10 mg/kg) animals (*p* < 0.05; Figure 5B). The lower dose of THIP pretreatment (5 mg/kg) also reduced seizure score but not reaching statistical significance. Therefore, THIP may be used as a potential anticonvulsant drug to suppress seizure behaviors in living animals.

### Tonic inhibition and basal GABAergic neurotransmission

**Figure 6:**
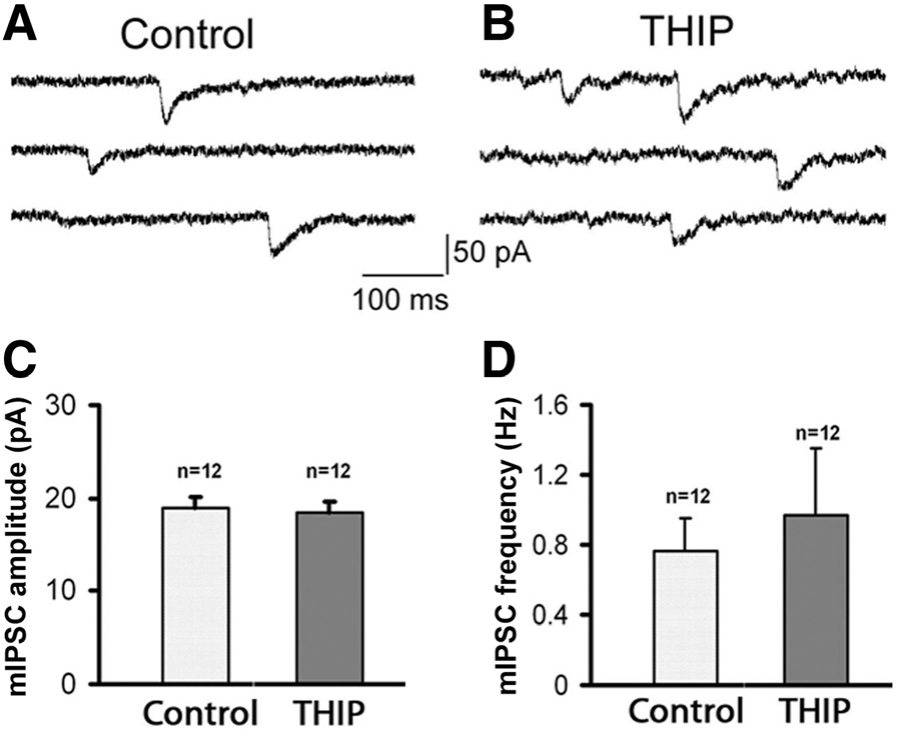
**THIP at low concentration has no effect on the amplitude and frequency of mIPSCs. A**, **B**, Representative traces showing mIPSCs recorded in control and THIP (5 μM) treated neurons. Note that in the presence of THIP, baseline was always noisier than control traces, indicating the activation of extrasynaptic GABA_A_-Rs. **C**, **D**, No significant difference between the mIPSC amplitude (*p* > 0.6) and frequency (*p* > 0.4) in control and THIP-treated neurons.

While previous studies have linked tonic inhibition with seizure threshold [31,44], the underlying mechanism is not fully understood. We decided to investigate the mechanism of tonic inhibition in regulating epileptiform activity. One question we addressed is whether tonic inhibition has any direct effect on basal GABAergic synaptic transmission in rat hippocampal cultures. Spontaneous miniature inhibitory postsynaptic currents (mIPSCs) were recorded in the presence of TTX (1 μM) and CNQX (10 μM) to block action potentials and glutamatergic responses, respectively. We have previously shown that THIP at micromolar concentrations elicited a tonic current (10–30 pA) in hippocampal neurons [34]. Here, we found that application of 5 μM THIP had no significant effect on both the mIPSC amplitude (control, 18.9 ± 1.1 pA, n = 12; THIP, 18.3 ± 1.3 pA, n = 12; p > 0.6) and the frequency (control, 0.76 ± 0.18 Hz, n = 12; THIP, 0.96 ± 0.30 Hz, n = 12; p > 0.4) (Figure 6). We did notice that the baseline noise in the presence of THIP was always larger than the controls, indicating the tonic activation of extrasynaptic GABA_A_-Rs by low concentration of THIP. The null effect of 5 μM THIP on mIPSCs suggested that synaptic GABA_A_-Rs are not significantly affected at this low concentration of THIP.

### Mechanism of tonic inhibition in modulating neuronal activity

**Figure 7:**
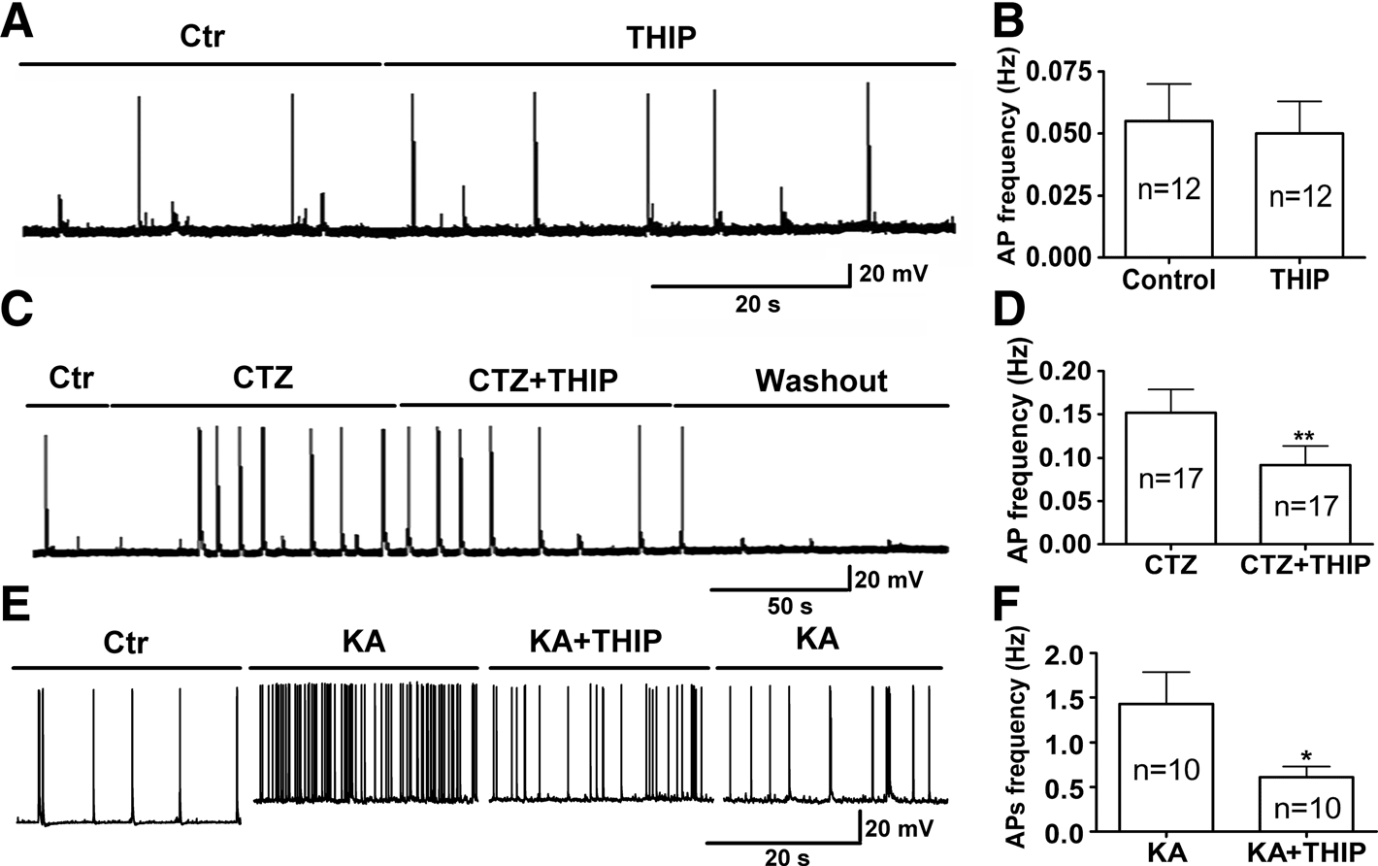
**THIP effect on basal and elevated neuronal activity. A**, **B**, THIP had no significant effect on the basal level of action potential firing (*p* > 0.4, n = 12). **C**, **D**, THIP significantly inhibited CTZ-induced action potentials (*p* < 0.01, n = 17). **E**, **F**, THIP also inhibited KA-induced action potentials (*p* < 0.03, n = 10).

We next examined the effect of THIP on neuronal activity at basal level as well as under stimulated conditions. In normal bathing solution, application of low concentration of THIP (5 μM) showed no significant effect on action potential firing in the majority of neurons tested (Figure 7A-B; control, 0.055 ± 0.015 Hz; THIP, 0.050 ± 0.013 Hz; n = 12, *p* > 0.4, paired *t* test). However, when neuronal activity was elevated by CTZ (5 μM) as demonstrated previously [36], THIP exerted a significant inhibitory effect on neuronal activity (Figure 7C-D). Action potential firing frequency dropped significantly from 0.152 ± 0.027 Hz under CTZ application to 0.092 ± 0.022 Hz when THIP was applied together with CTZ (n = 17, *p* < 0.01, paired *t* test). To confirm that THIP has greater effect when neuronal activity is elevated, we further stimulated neurons with low concentration of kainic acid (KA). Bath application of KA (2.5 μM) induced a large membrane depolarization and a remarkable increase of action potential firing (Figure 7E). Importantly, co-application of 5 μM THIP together with KA significantly decreased the firing frequency in all of the neurons recorded (Figure 7E-F; KA, 1.4 ± 0.4 Hz; KA+THIP, 0.6 ± 0.1 Hz; n = 10, *p* < 0.03, paired *t* test). Together, our results suggest that tonic inhibition may have greater modulatory effect on neural networks with hyperexcitatory activity, such as that under epileptogenic stimulation, making extrasynaptic GABA_A_-Rs ideal for developing novel anti-convulsant drugs.

## Discussion

In this study, we have demonstrated that two distinct subtypes of extrasynaptic GABA_A_-Rs both play an important role in regulating the formation of epileptiform activity in hippocampal cultures. Enhancing tonic inhibition in living animals *in vivo* also modulates epileptiform activity and behavioral seizures in a dose-dependent manner. More importantly, we demonstrated that tonic inhibition potently inhibits high frequency action potentials under stimulated conditions but not at basal low frequency firing condition, suggesting a novel mechanism of tonic inhibition in regulating neural network activity.

### Extrasynaptic GABA_A_-Rs regulate epileptogenesis

Due to its continuous activation by ambient GABA, the charge transfer of tonic currents mediated by extrasynaptic GABA_A_-Rs have been estimated to be several folds more than phasic currents mediated by synaptic GABA_A_-Rs [4,45,46]. Tonic GABA conductance controls the overall gain of neuronal input–output [5,45-48]. Therefore, when GABA_A_ receptor α5 and δ subunits were found significantly reduced in the hippocampus of animal TLE models [8,25,49,50], it was realized that downregulation of tonic inhibition might have contributed to epileptogenesis. Consistently, our previous work has also demonstrated that tonic GABA inhibition was downregulated after chronic epileptogenic stimulation in cultured hippocampal neurons [34]. However, later studies found that despite a reduction of α5 and δ subunit expression in epileptic mice, tonic inhibition in the hippocampus was largely maintained or even increased, possibly mediated by increased expression of α4γ2-containing GABA_A_-Rs [26,28,32]. Thus, tonic inhibition might have undergone homeostatic changes during and after epileptogenesis [51]. Our current study demonstrated that enhancing tonic inhibition by increasing the expression level of either α5β3γ2 or α6β3δ receptors can effectively suppress epileptiform activity. We have further demonstrated that seizure behaviors are attenuated by enhancing tonic inhibition *in vivo*. Based on previous and our own studies, we attribute an important role to tonic inhibition in modulating hippocampal epileptogenesis: enhancing tonic inhibition will inhibit epileptiform activity, while reducing tonic inhibition will increase the susceptibility of epileptic seizures [31,44].

### Overexpression of extrasynaptic GABA_A_-Rs regulates epileptiform activity

While THIP has been used previously to modulate epileptiform activity, the target receptors were usually not specifically identified because higher concentrations of THIP might activate different combinations of synaptic and extrasynaptic GABA_A_-Rs. Our current study provides more direct evidence on extrasynaptic regulation of epileptogenesis by demonstrating that overexpression of both α5β3γ2 and α6β3δ receptors can effectively attenuate epileptiform activity. We showed that neurons transfected with both α5β3γ2 and α6β3δ receptors had enhanced tonic currents compared to control neurons. Mutations in the δ subunit of GABA_A_ receptors have been mapped in human epilepsy patients [29,30], indicating the clinical relevance of δ-GABA_A_ receptors. Our molecular expression studies suggest that extrasynaptic GABA_A_-Rs may be a potential therapeutic target for developing antiepileptic drugs to treat TLE.

### THIP regulation of neuronal activity

Previous studies have reported that THIP may inhibit neuronal activity [52-56]. However, these studies used concentrations much higher than our current work. It is known that high concentration of THIP may directly activate γ2-containing synaptic GABA_A_-Rs [42,57,58]. In this study, we used a low concentration of THIP (5 μM) that did not affect mIPSCs, indicating that at this concentration THIP did not activate synaptic GABA_A_-Rs. Correspondingly, we found that 5 μM THIP did not affect basal neuronal firing in the majority of neurons tested. More importantly, we discovered a strong inhibitory effect of THIP on elevated neuronal activity induced by both CTZ and KA. It is possible that elevated neuronal activity may induce substantial release of GABA [46], which will act together with THIP to enhance tonic inhibition and reduce neuronal activity. Another possibility is that tonic current may be outward rectifying at depolarized membrane potential [59], making the effect of THIP more potent when neurons are hyperexcitatory. Our discovery of the preferential inhibition of THIP on elevated activity makes it an ideal candidate for anticonvulsant drug, because it may have less side effects comparing to those affecting basal neural activity.

## Conclusions

We employed both molecular and pharmacological tools to demonstrate that tonic inhibition modulates epileptiform activity both *in vitro* and *in vivo*. The overexpression of both α5β3γ2 and α6β3δ receptors inhibited the formation of epileptiform activity in hippocampal neurons, establishing unambiguously a solid ground for extrasynaptic modulation of epileptogenesis. Furthermore, we discovered a more prominent role of tonic inhibition in inhibiting hyperexcitatory activity rather than low frequency basal activity, suggesting that extrasynaptic GABA_A_-Rs are ideal drug targets for developing anti-convulsant drugs that may specifically act against epileptiform activity without much side effect on normal brain functions.

## Methods

### Primary neuronal culture

Primary hippocampal neurons were prepared from embryonic day 18 Sprague–Dawley rat embryos of either sex, similar to our previous work with modifications [34]. Briefly, after dissection of the hippocampi, the tissue was rinsed in cold HBS and then digested with 0.05% trypsin-EDTA for 20 min at 37°C, followed by trituration with pipettes in the plating medium (DMEM with 10% FBS and 10% F12). After rinsing for twice, cells were counted and plated onto coverslips precoated with 0.1 mg/ml poly-D-lysine (Sigma). After culturing for 1 day, media were changed into neuronal culture media (neurobasal media containing 2 mM GlutaMAX™-I Supplement and 2% B-27). AraC (1 μM, Sigma) was added 6–8 days after plating, and cells were fed twice weekly thereafter and maintained at 37°C and in 5% CO_2_ incubators. Trypsin-EDTA, DMEM, FBS, F12, Neurobasal media, GlutaMAX™-I Supplement and B-27 were purchased from Invitrogen Corporation. Some experiments were also performed using mouse hippocampal cultures.

### DNA transfection

Calcium-phosphate transfection was performed similar to the protocol previously described [60]. Neurons were transfected at 10 days *in vitro* (DIV). The plasmids of rat α5, α6, β3, γ2, δ subunits of GABA_A_ receptors (gifts from Drs. Robert Macdonald, Matthias Kneussel, and Dr. Bernhard Luscher) were co-transfected with pEGFP or mCherry (Clontech). Transfection with EGFP or mCherry alone served as controls. Most of the experiments were performed around 2 weeks of culture unless otherwise indicated.

### Electrophysiological recordings in cultured hippocampal neurons

Whole-cell recordings were performed in current- or voltage-clamp mode using a MultiClamp 700B amplifier (Axon Instruments). Patch pipettes were pulled from borosilicate glass (Sutter Instrument, BF150-86-10) and fire polished (4–6 MΩ). The recording chamber was continuously perfused with a bath solution consisting of (mM): 128 NaCl, 30 Glucose, 25 Hepes, 5 KCl, 2 CaCl_2_, 1 MgCl_2_, pH 7.3 adjusted with NaOH. The pipette solution for recording action potentials and mEPSCs contained (mM): 125 K-gluconate, 10 KCl, 5 EGTA, 10 Hepes, 10 Tris-phosphocreatine, 4 MgATP, 0.5 NaGTP, pH 7.3 adjusted with KOH. For tonic GABA currents and GABA-induced whole-cell currents, patch pipettes were filled with (mM): 135 KCl, 10 Tris-phosphocreatine, 2 EGTA, 10 Hepes, 4 MgATP, 0.5 NaGTP, pH 7.3 adjusted with KOH. Liquid junction potentials were always corrected before forming giga-ohm seal. The series resistance was typically 10–20 MΩ and partially compensated by 30-50%. Data were acquired using pClamp 10.2 software (Axon Instruments), sampled at 2–10 kHz, and filtered at 1 kHz. Off-line analysis was done with Clampfit 10.2 software (Axon Instruments). Miniature events were analyzed using Mini Analysis software (Synaptosoft). Large depolarization shift resembling paroxysmal depolarization shift is defined here as ≥ 10 mV depolarization and ≥ 300 ms in duration. An epileptiform burst is defined by at least five consecutive action potentials overlaying on top of the large depolarization shift. When quantifying the percentage of neurons showing epileptiform activity, the criterion is at least two epileptiform bursts occurring during 10 min of recording. All of the drugs used were freshly diluted in bath solution to their final concentrations before experiments.

### Electrophysiological recordings in anaesthetized rats

Adult male Sprague–Dawley rats weighing between 250–350 g were maintained on an *ad libitum* feeding schedule and kept on a 12 hr on/off light cycle. During electrophysiological study, rats were anesthetized with urethane (1.2 g/kg, i.p.) and the level of anesthesia was assessed by the absence of a withdrawal reflex, and additional anesthetic (urethane, 0.2-0.6 mg/kg, i.p.) was administered as necessary. Body temperature was maintained at 37 ± 0.5°C with a Harvard Homoeothermic Blanket (Harvard Apparatus Limited, Kent, UK). At the end of experiments, animals were killed with an overdose of urethane. All animal experiments were approved by the local committee of Laboratory Animals, Fudan University and carried out in accordance with Chinese National Science Foundation animal research regulation. Animal preparation was similar to previously reported [36,37,39]. Briefly, all the animals had their lateral tail vein cannulated for drug administration and then mounted in a stereotaxic frame. An incision was made in the midline of the head to expose the top part of the skull for the implantation of i.c.v. guide cannula (22GA, Plastics One, USA) into the lateral ventricle (0.3 mm posterior to bregma, 1.3 mm lateral to the midline, and 4 mm below the skull surface), and then secured by the dental cement. For recording and stimulating, a large burr hole was made in the left side of the incised skull above the hippocampal area, and the dura was pierced and removed. A concentric bipolar stimulating electrode (Harvard Apparatus) was placed close to the CA3 region (3.8-4.5 mm posterior to bregma, 3.5-4.0 mm lateral to the midline, and 3.0-3.8 mm below the brain surface) in order to stimulate the Shaffer collateral pathway. For recording in the CA1 pyramidal cell layer, a tungsten electrode (0.5 MΩ, WPI, USA) was placed 3.5-4.2 mm posterior to bregma, 2.0-3.0 mm lateral to the midline. The depth of the recording electrode was approximately 2.0-2.5 mm below the brain surface as determined by the sudden change of electrical noise and the shape of the evoked field excitatory postsynaptic potentials (fEPSPs) and population spike (PS). For stimulation, a constant current generator passed a square-wave pulse (0.2 ms in duration) through the stimulating electrode (test pulse) and the stimulation frequency was set at once per minute. The electrophysiological signals were amplified and filtered (0.3-3 kHz) using a NeuroLog System (Digitimer Ltd., Hearts, UK) and visualized and stored in a PC computer through an A-D converter, CED 1401 micro (Cambridge Electronic Design, Cambridge, UK). After both electrodes were in the right place, the fEPSPs and PS were monitored for at least 30 min until a stable recording was achieved. Following a 30 min baseline recording, CTZ (5 μmol, 5 μL) was administered i.c.v. via the pre-implanted guide cannula into the left lateral ventricle. Pharmacologically induced seizure-like activity was monitored after CTZ injection by observing the change of the evoked potentials transforming from single PS into a multi-peaked display, and spontaneous seizure burst activity in CA1 pyramidal neurons [37,39,61]. After the epileptiform burst activity was stable for at least 30 min, THIP (4 mg/kg in 1 mL/kg) or vehicles were delivered through the cannula pre-implanted in the lateral tail vein. To confirm correct placement of the electrode and cannula, the brain was taken for histological validation of the injection and recording/stimulating sites. Epileptiform activity within CA1 pyramidal cells was analyzed offline using Spike2 software (an analyzing program for CED 1401, Cambridge Electronics, UK) and specific scripts designed for this study with Spike2. The highly synchronized bursting activity was defined as having high frequency multiple high amplitude spikes (>0.5 mV) with an initial interspike interval of less than 0.1 s, a minimum of 5 spikes, and burst duration over 1 s [37].

### Behavioral test in freely moving rats

CTZ induced seizure behavioral test was carried out similarly as previously reported [38]. Briefly, under general anesthetics with sodium pentobarbital (60 mg/kg, i.p.), a guide cannula was pre-implanted into left lateral ventricle (0.3 mm posterior to bregma, 1.3 mm lateral to the midline, and 4 mm below the skull surface) at least 5 days before the behavioral test. Cannula-implanted animals were randomly divided into following experimental groups: 1) CTZ group: 0.25 μmol (i.c.v.) for one injection per day, three consecutive days; 2) THIP + CTZ group one: 5 mg/kg (i.p.) THIP + 0.25 μmol (i.c.v.) CTZ for one injection per day, three consecutive days; 3) THIP + CTZ group two: 10 mg/kg (i.p.) THIP + 0.25 μmol (i.c.v.) CTZ for one injection per day, three consecutive days. All behavioral tests were carried out between 2:00 pm and 7:00 pm. The animals were first placed in a plastic cage and acclimatized for at least half an hour before experiments. Before and after drug injection, animal behavior was continuously monitored for a period of 1 and 3 hours with video recording, respectively. Behavioral seizures were scored using 5-graded Racine Score system [62]. Briefly, Racine score I, facial clonus; score II, head nodding; score III, unilateral forelimb clonus; score IV, rearing with bilateral forelimb clonus; score V, rearing and falling (loss of postural control).

### Data analysis

Group data were expressed as mean ± SEM. Across different groups of data, statistical significance between means was determined using one-way ANOVA with Tukey HSD post hoc analysis. Comparison within a group used a paired or unpaired *t* test (GraphPad Prism, GraphPad Software Inc.). Pearson Chi-Square test was used for statistical analysis of percentage (SPSS). Significance level was set at *p* < 0.05.

### Drugs and solutions

Cyclothiazide (CTZ) and L655708 were purchased from Tocris (Northpoint, Bristol). THIP (4,5,6,7-tetrahydroisoxazolo[4,5-c] pyridine-3-ol and urethane (25% in distilled water) were purchased from Sigma Aldrich Chemical Co. (Poole, Dorset).

## Competing interest

The authors declare that they have no competing interests.

## Authors’ contributions

YS carried out most electrophysiological analysis in cultured neurons and in anesthetized animals. ZW and DJ performed experiments of overexpressing alpha5/beta3/gamma2 receptors in hippocampal cultures. SK analyzed THIP effect on animal seizure behaviors, and AP studied THIP effect on mIPSCs and epileptiform activity in cultured neurons. GC conceived and designed the project. GC and YS wrote the manuscript. YW supervised the in vivo and part of in vitro electrophysiology experiments and revised the manuscript. All authors read and approved the final manuscript.

## References

[B1] Duncan JS Sander JW Sisodiya SM Walker MC Adult epilepsy Lancet 2006 367 9516 1087 1100 10.1016/S0140-6736(06)68477-8 16581409

[B2] Treiman DM GABAergic mechanisms in epilepsy Epilepsia 2001 42 Suppl 3 8 12 11520315 10.1046/j.1528-1157.2001.042suppl.3008.x

[B3] Farrant M Nusser Z Variations on an inhibitory theme: phasic and tonic activation of GABA(A) receptors Nat Rev Neurosci 2005 6 3 215 229 10.1038/nrn1625 15738957

[B4] Mody I Pearce RA Diversity of inhibitory neurotransmission through GABA(A) receptors Trends Neurosci 2004 27 9 569 575 10.1016/j.tins.2004.07.002 15331240

[B5] Semyanov A Walker MC Kullmann DM Silver RA Tonically active GABA A receptors: modulating gain and maintaining the tone Trends Neurosci 2004 27 5 262 269 10.1016/j.tins.2004.03.005 15111008

[B6] Brunig I Scotti E Sidler C Fritschy JM Intact sorting, targeting, and clustering of gamma-aminobutyric acid A receptor subtypes in hippocampal neurons in vitro J Comp Neurol 2002 443 1 43 55 10.1002/cne.10102 11793346

[B7] Crestani F Keist R Fritschy JM Benke D Vogt K Prut L Bluthmann H Mohler H Rudolph U Trace fear conditioning involves hippocampal alpha5 GABA(A) receptors Proc Natl Acad Sci U S A 2002 99 13 8980 8985 10.1073/pnas.142288699 12084936 PMC124409

[B8] Houser CR Esclapez M Downregulation of the alpha5 subunit of the GABA(A) receptor in the pilocarpine model of temporal lobe epilepsy Hippocampus 2003 13 5 633 645 10.1002/hipo.10108 12921352

[B9] Caraiscos VB Elliott EM You-Ten KE Cheng VY Belelli D Newell JG Jackson MF Lambert JJ Rosahl TW Wafford KA MacDonald JF Orser BA Tonic inhibition in mouse hippocampal CA1 pyramidal neurons is mediated by alpha5 subunit-containing gamma-aminobutyric acid type A receptors Proc Natl Acad Sci U S A 2004 101 10 3662 3667 10.1073/pnas.0307231101 14993607 PMC373519

[B10] Serwanski DR Miralles CP Christie SB Mehta AK Li X De Blas AL Synaptic and nonsynaptic localization of GABAA receptors containing the alpha5 subunit in the rat brain J Comp Neurol 2006 499 3 458 470 10.1002/cne.21115 16998906 PMC2749292

[B11] Wei W Zhang N Peng Z Houser CR Mody I Perisynaptic localization of delta subunit-containing GABA(A) receptors and their activation by GABA spillover in the mouse dentate gyrus J Neurosci 2003 23 33 10650 10661 14627650 10.1523/JNEUROSCI.23-33-10650.2003 PMC6740905

[B12] Nusser Z Sieghart W Somogyi P Segregation of different GABAA receptors to synaptic and extrasynaptic membranes of cerebellar granule cells J Neurosci 1998 18 5 1693 1703 9464994 10.1523/JNEUROSCI.18-05-01693.1998 PMC6792611

[B13] Stell BM Brickley SG Tang CY Farrant M Mody I Neuroactive steroids reduce neuronal excitability by selectively enhancing tonic inhibition mediated by delta subunit-containing GABAA receptors Proc Natl Acad Sci U S A 2003 100 24 14439 14444 10.1073/pnas.2435457100 14623958 PMC283610

[B14] Casula MA Bromidge FA Pillai GV Wingrove PB Martin K Maubach K Seabrook GR Whiting PJ Hadingham KL Identification of amino acid residues responsible for the alpha5 subunit binding selectivity of L-655,708, a benzodiazepine binding site ligand at the GABA(A) receptor J Neurochem 2001 77 2 445 451 10.1046/j.1471-4159.2001.00289.x 11299307

[B15] Atack JR Bayley PJ Seabrook GR Wafford KA McKernan RM Dawson GR L-655,708 enhances cognition in rats but is not proconvulsant at a dose selective for alpha5-containing GABAA receptors Neuropharmacology 2006 51 6 1023 1029 10.1016/j.neuropharm.2006.04.018 17046030

[B16] Nusser Z Mody I Selective modulation of tonic and phasic inhibitions in dentate gyrus granule cells J Neurophysiol 2002 87 5 2624 2628 11976398 10.1152/jn.2002.87.5.2624

[B17] Cope DW Hughes SW Crunelli V GABAA receptor-mediated tonic inhibition in thalamic neurons J Neurosci 2005 25 50 11553 11563 10.1523/JNEUROSCI.3362-05.2005 16354913 PMC6726040

[B18] Wohlfarth KM Bianchi MT Macdonald RL Enhanced neurosteroid potentiation of ternary GABA(A) receptors containing the delta subunit J Neurosci 2002 22 5 1541 1549 11880484 10.1523/JNEUROSCI.22-05-01541.2002 PMC6758857

[B19] Belelli D Harrison NL Maguire J Macdonald RL Walker MC Cope DW Extrasynaptic GABAA receptors: form, pharmacology, and function J Neurosci 2009 29 41 12757 12763 10.1523/JNEUROSCI.3340-09.2009 19828786 PMC2784229

[B20] Sperk G Furtinger S Schwarzer C Pirker S GABA and its receptors in epilepsy Adv Exp Med Biol 2004 548 92 103 10.1007/978-1-4757-6376-8_7 15250588

[B21] Mann EO Mody I The multifaceted role of inhibition in epilepsy: seizure-genesis through excessive GABAergic inhibition in autosomal dominant nocturnal frontal lobe epilepsy Curr Opin Neurol 2008 21 2 155 160 10.1097/WCO.0b013e3282f52f5f 18317273

[B22] Coulter DA Carlson GC Functional regulation of the dentate gyrus by GABA-mediated inhibition Prog Brain Res 2007 163 235 243 17765722 10.1016/S0079-6123(07)63014-3

[B23] Sierra-Paredes G Sierra-Marcuno G Extrasynaptic GABA and glutamate receptors in epilepsy CNS Neurol Disord Drug Targets 2007 6 4 288 300 10.2174/187152707781387251 17691986

[B24] Richerson GB Looking for GABA in all the wrong places: the relevance of extrasynaptic GABA(A) receptors to epilepsy Epilepsy Curr 2004 4 6 239 242 10.1111/j.1535-7597.2004.46008.x 16059513 PMC1176385

[B25] Peng Z Huang CS Stell BM Mody I Houser CR Altered expression of the delta subunit of the GABAA receptor in a mouse model of temporal lobe epilepsy J Neurosci 2004 24 39 8629 8639 10.1523/JNEUROSCI.2877-04.2004 15456836 PMC6729896

[B26] Zhang N Wei W Mody I Houser CR Altered localization of GABA(A) receptor subunits on dentate granule cell dendrites influences tonic and phasic inhibition in a mouse model of epilepsy J Neurosci 2007 27 28 7520 7531 10.1523/JNEUROSCI.1555-07.2007 17626213 PMC6672608

[B27] Goodkin HP Joshi S Mtchedlishvili Z Brar J Kapur J Subunit-specific trafficking of GABA(A) receptors during status epilepticus J Neurosci 2008 28 10 2527 2538 10.1523/JNEUROSCI.3426-07.2008 18322097 PMC2880323

[B28] Rajasekaran K Joshi S Sun C Mtchedlishvilli Z Kapur J Receptors with low affinity for neurosteroids and GABA contribute to tonic inhibition of granule cells in epileptic animals Neurobiol Dis 2010 40 2 490 501 10.1016/j.nbd.2010.07.016 20682339 PMC2940226

[B29] Dibbens LM Feng HJ Richards MC Harkin LA Hodgson BL Scott D Jenkins M Petrou S Sutherland GR Scheffer IE Berkovic SF Macdonald RL Mulley JC GABRD encoding a protein for extra- or peri-synaptic GABAA receptors is a susceptibility locus for generalized epilepsies Hum Mol Genet 2004 13 13 1315 1319 10.1093/hmg/ddh146 15115768

[B30] Feng HJ Kang JQ Song L Dibbens L Mulley J Macdonald RL Delta subunit susceptibility variants E177A and R220H associated with complex epilepsy alter channel gating and surface expression of alpha4beta2delta GABAA receptors J Neurosci 2006 26 5 1499 1506 10.1523/JNEUROSCI.2913-05.2006 16452673 PMC6675478

[B31] Maguire JL Stell BM Rafizadeh M Mody I Ovarian cycle-linked changes in GABA(A) receptors mediating tonic inhibition alter seizure susceptibility and anxiety Nat Neurosci 2005 8 6 797 804 10.1038/nn1469 15895085

[B32] Scimemi A Semyanov A Sperk G Kullmann DM Walker MC Multiple and plastic receptors mediate tonic GABAA receptor currents in the hippocampus J Neurosci 2005 25 43 10016 10024 10.1523/JNEUROSCI.2520-05.2005 16251450 PMC6725560

[B33] Cope DW Di Giovanni G Fyson SJ Orban G Errington AC Lorincz ML Gould TM Carter DA Crunelli V Enhanced tonic GABAA inhibition in typical absence epilepsy Nat Med 2009 15 12 1392 1398 10.1038/nm.2058 19966779 PMC2824149

[B34] Qi JS Yao J Fang C Luscher B Chen G Downregulation of tonic GABA currents following epileptogenic stimulation of rat hippocampal cultures J Physiol 2006 577 Pt 2 579 590 16990405 10.1113/jphysiol.2006.113134 PMC1890447

[B35] Bai D Zhu G Pennefather P Jackson MF MacDonald JF Orser BA Distinct functional and pharmacological properties of tonic and quantal inhibitory postsynaptic currents mediated by gamma-aminobutyric acid(A) receptors in hippocampal neurons Mol Pharmacol 2001 59 4 814 824 11259626 10.1124/mol.59.4.814

[B36] Qi J Wang Y Jiang M Warren P Chen G Cyclothiazide induces robust epileptiform activity in rat hippocampal neurons both in vitro and in vivo J Physiol 2006 571 Pt 3 605 618 16423850 10.1113/jphysiol.2005.103812 PMC1805799

[B37] Wang Y Qi JS Kong S Sun Y Fan J Jiang M Chen G BDNF-TrkB signaling pathway mediates the induction of epileptiform activity induced by a convulsant drug cyclothiazide Neuropharmacology 2009 57 1 49 59 10.1016/j.neuropharm.2009.04.007 19393251 PMC2733837

[B38] Kong S Qian B Liu J Fan M Chen G Wang Y Cyclothiazide induces seizure behavior in freely moving rats Brain Res 2010 1355 207 213 20678492 10.1016/j.brainres.2010.07.088 PMC2947190

[B39] Qian B Sun Y Wu Z Wan L Chen L Kong S Zhang B Zhang F Wang ZY Wang Y Epileptiform response of CA1 neurones to convulsant stimulation by cyclothiazide, kainic acid and pentylenetetrazol in anaesthetized rats Seizure 2011 20 312 319 10.1016/j.seizure.2010.12.016 21269843

[B40] Lindquist CE Ebert B Birnir B Extrasynaptic GABA(A) channels activated by THIP are modulated by diazepam in CA1 pyramidal neurons in the rat brain hippocampal slice Mol Cell Neurosci 2003 24 1 250 257 10.1016/S1044-7431(03)00128-3 14550784

[B41] Krogsgaard-Larsen P Frolund B Liljefors T Ebert B GABA(A) agonists and partial agonists: THIP (Gaboxadol) as a non-opioid analgesic and a novel type of hypnotic Biochem Pharmacol 2004 68 8 1573 1580 10.1016/j.bcp.2004.06.040 15451401

[B42] Brown N Kerby J Bonnert TP Whiting PJ Wafford KA Pharmacological characterization of a novel cell line expressing human alpha(4)beta(3)delta GABA(A) receptors Br J Pharmacol 2002 136 7 965 974 10.1038/sj.bjp.0704795 12145096 PMC1573424

[B43] Wafford KA Ebert B Gaboxadol–a new awakening in sleep Curr Opin Pharmacol 2006 6 1 30 36 10.1016/j.coph.2005.10.004 16368265

[B44] Spigelman I Li Z Banerjee PK Mihalek RM Homanics GE Olsen RW Behavior and physiology of mice lacking the GABAA-receptor delta subunit Epilepsia 2002 43 Suppl 5 3 8 12121286 10.1046/j.1528-1157.43.s.5.8.x

[B45] Cavelier P Hamann M Rossi D Mobbs P Attwell D Tonic excitation and inhibition of neurons: ambient transmitter sources and computational consequences Prog Biophys Mol Biol 2005 87 1 3 16 10.1016/j.pbiomolbio.2004.06.001 15471587 PMC8906495

[B46] Glykys J Mody I Activation of GABAA receptors: views from outside the synaptic cleft Neuron 2007 56 5 763 770 10.1016/j.neuron.2007.11.002 18054854

[B47] Mitchell SJ Silver RA Shunting inhibition modulates neuronal gain during synaptic excitation Neuron 2003 38 3 433 445 10.1016/S0896-6273(03)00200-9 12741990

[B48] Chadderton P Margrie TW Hausser M Integration of quanta in cerebellar granule cells during sensory processing Nature 2004 428 6985 856 860 10.1038/nature02442 15103377

[B49] Fritschy JM Kiener T Bouilleret V Loup F GABAergic neurons and GABA(A)-receptors in temporal lobe epilepsy Neurochem Int 1999 34 5 435 445 10.1016/S0197-0186(99)00040-6 10397372

[B50] Houser CR Esclapez M Vulnerability and plasticity of the GABA system in the pilocarpine model of spontaneous recurrent seizures Epilepsy Res 1996 26 1 207 218 10.1016/S0920-1211(96)00054-X 8985701

[B51] Mody I Aspects of the homeostaic plasticity of GABAA receptor-mediated inhibition J Physiol 2005 562 Pt 1 37 46 15528237 10.1113/jphysiol.2004.077362 PMC1665492

[B52] Krook-Magnuson EI Li P Paluszkiewicz SM Huntsman MM Tonically active inhibition selectively controls feedforward circuits in mouse barrel cortex J Neurophysiol 2008 100 2 932 944 10.1152/jn.01360.2007 18509076 PMC2525715

[B53] Gao H Smith BN Tonic GABAA receptor-mediated inhibition in the rat dorsal motor nucleus of the vagus J Neurophysiol 2010 103 2 904 914 10.1152/jn.00511.2009 20018836 PMC2822683

[B54] Olmos-Serrano JL Paluszkiewicz SM Martin BS Kaufmann WE Corbin JG Huntsman MM Defective GABAergic neurotransmission and pharmacological rescue of neuronal hyperexcitability in the amygdala in a mouse model of fragile X syndrome J Neurosci 2010 30 29 9929 9938 10.1523/JNEUROSCI.1714-10.2010 20660275 PMC2948869

[B55] Edwards MD White AM Platt B Characterisation of rat superficial superior colliculus neurones: firing properties and sensitivity to GABA Neuroscience 2002 110 1 93 104 10.1016/S0306-4522(01)00558-9 11882375

[B56] Judge SJ Ingram CD Gartside SE GABA receptor modulation of 5-HT neuronal firing: characterization and effect of moderate in vivo variations in glucocorticoid levels Neurochem Int 2004 45 7 1057 1065 10.1016/j.neuint.2004.05.003 15337305

[B57] Storustovu SI Ebert B Pharmacological characterization of agonists at delta-containing GABAA receptors: Functional selectivity for extrasynaptic receptors is dependent on the absence of gamma2 J Pharmacol Exp Ther 2006 316 3 1351 1359 16272218 10.1124/jpet.105.092403

[B58] Cremers T Ebert B Plasma and CNS concentrations of Gaboxadol in rats following subcutaneous administration Eur J Pharmacol 2007 562 1–2 47 52 17362924 10.1016/j.ejphar.2007.01.017

[B59] Pavlov I Savtchenko LP Kullmann DM Semyanov A Walker MC Outwardly rectifying tonically active GABAA receptors in pyramidal cells modulate neuronal offset, not gain J Neurosci 2009 29 48 15341 15350 10.1523/JNEUROSCI.2747-09.2009 19955387 PMC6665960

[B60] Jiang M Chen G High Ca2+−phosphate transfection efficiency in low-density neuronal cultures Nat Protoc 2006 1 2 695 700 10.1038/nprot.2006.86 17406298

[B61] Wheal HV Bernard C Chad JE Cannon RC Pro-epileptic changes in synaptic function can be accompanied by pro-epileptic changes in neuronal excitability Trends Neurosci 1998 21 4 167 174 10.1016/S0166-2236(97)01182-X 9554727

[B62] Racine RJ Modification of seizure activity by electrical stimulation. II. Motor seizure Electroencephalogr Clin Neurophysiol 1972 32 3 281 294 10.1016/0013-4694(72)90177-0 4110397

